# Anthropometric Indices With Insulin Resistance in Obese Patients: A Literature Review

**DOI:** 10.7759/cureus.41881

**Published:** 2023-07-14

**Authors:** Khalid Khan, Anil Wanjari, Sourya Acharya, Sabiha Quazi

**Affiliations:** 1 Department of Medicine, Jawaharlal Nehru Medical College, Datta Meghe Institute of Higher Education and Research (DMIHER), Wardha, IND; 2 Department of Dermatology, Jawaharlal Nehru Medical College, Datta Meghe Institute of Higher Education and Research (DMIHER), Wardha, IND

**Keywords:** waist circumference, body mass index, anthropometric parameter, insulin resistance, obesity

## Abstract

The hormone insulin is responsible for regulating the metabolism of proteins, carbs, and lipids by promoting the absorption of molecules such as glucose from the bloodstream into fat, the liver, and skeletal muscle cells. Insulin resistance (IR) is considered to be a physiological response to obesity that inhibits fat from accumulating and supports weight stabilization. People with IR gain less weight than those with insulin sensitivity, and therefore IR individuals have a three-fold increased likelihood of losing more weight when compared with insulin-sensitive individuals. A person's health is jeopardized by obesity, which is defined as excessive or unusual storage of fat in adipose tissue. Early identification using different anthropometric measuring parameters and proper and suitable therapy is essential as the incidence of obesity cases is increasing as a result of sedentary lifestyles, bad eating habits, a lack of physical exercise, and a lack of knowledge among young adults. The review followed the Preferred Reporting Items for Systematic Reviews and Meta-Analyses (PRISMA) guidelines, following which based on inclusion and exclusion criteria, eight articles were considered for the review. The analysis showed that all the parameters are easily accessible and hence can be used in daily practices. Due to being readily available, body mass index (BMI) and waist circumference (WC) constituted the most often employed anthropometric measures in everyday practices. In addition, variances in the values of the variables were seen due to differences in gender.

## Introduction and background

Insulin is a hormone that stimulates the uptake of substances such as glucose from the circulation into fat, the liver, and skeletal muscle cells, controlling the metabolism of proteins, carbohydrates, and lipids. Resistance to insulin is brought on by a decrease in insulin signaling, particularly in the insulin receptor substrate/phosphoinositide-3-kinase /protein kinase B axis, which may impact insulin's metabolic effects. Adult obesity and excess weight are recognized as the likelihood of having cardiovascular disease, type 2 diabetes, and cancers linked to being excess weight. These findings demonstrate a strong association between adult weight gain and rising insulin resistance (IR) discovered by multiple investigators [1]. IR is thought to be a bodily reaction to being overweight that prevents the accumulation of fat and promotes weight stabilization. IR causes people to gain less weight in comparison to insulin-sensitive individuals [2]. Obesity is termed as an excessive or unusual storing of fat in adipose tissue, which compromises a person's well-being [3-5]. IR is the frequently known theory to describe the cause and mechanism of metabolic syndrome [6,7].

Anthropometric indices used to evaluate obesity or cardiovascular risk or metabolic syndrome are body roundness index (BRI), weight, waist-to-hip ratio (WHR), body mass index (BMI), height, hip circumference (HC), neck circumference (NC), waist to height ratio (WHtR), conicity index, abdominal volume index (AVI), a body shape index (ABSI), waist circumference (WC), body adiposity index (BAI), and visceral adiposity index (VAI) [8-14]. The basic mechanism behind these anthropometric derangements is obesity and dyslipidemia secondary to IR [6,7]. Various indirect markers of IR are homeostasis model assessment for IR (HOMA-IR), quantitative insulin sensitivity check index (QUICKI), triglyceride/high-density lipoprotein cholesterol (TG/HDL-C) or (TG/HDL) ratio, glucose-to-insulin ratio, fasting IR index, the logarithm of HOMA-IR, fasting lipid profile (FLP), fasting blood sugar (FBS), and fasting insulin level [15-17].

Therefore, the relation between obesity (measured by anthropometric indices) and IR is of great importance. Assessment of obesity by anthropometric measurement and indices is the cheapest available tool to the physician. However, a variety of anthropometric indices are available, and it is not possible for one to do all in routine practice. Hence, this review is conducted to compare the various anthropometric parameters and their specificity.

## Review

Search methodology

**Figure 1:**
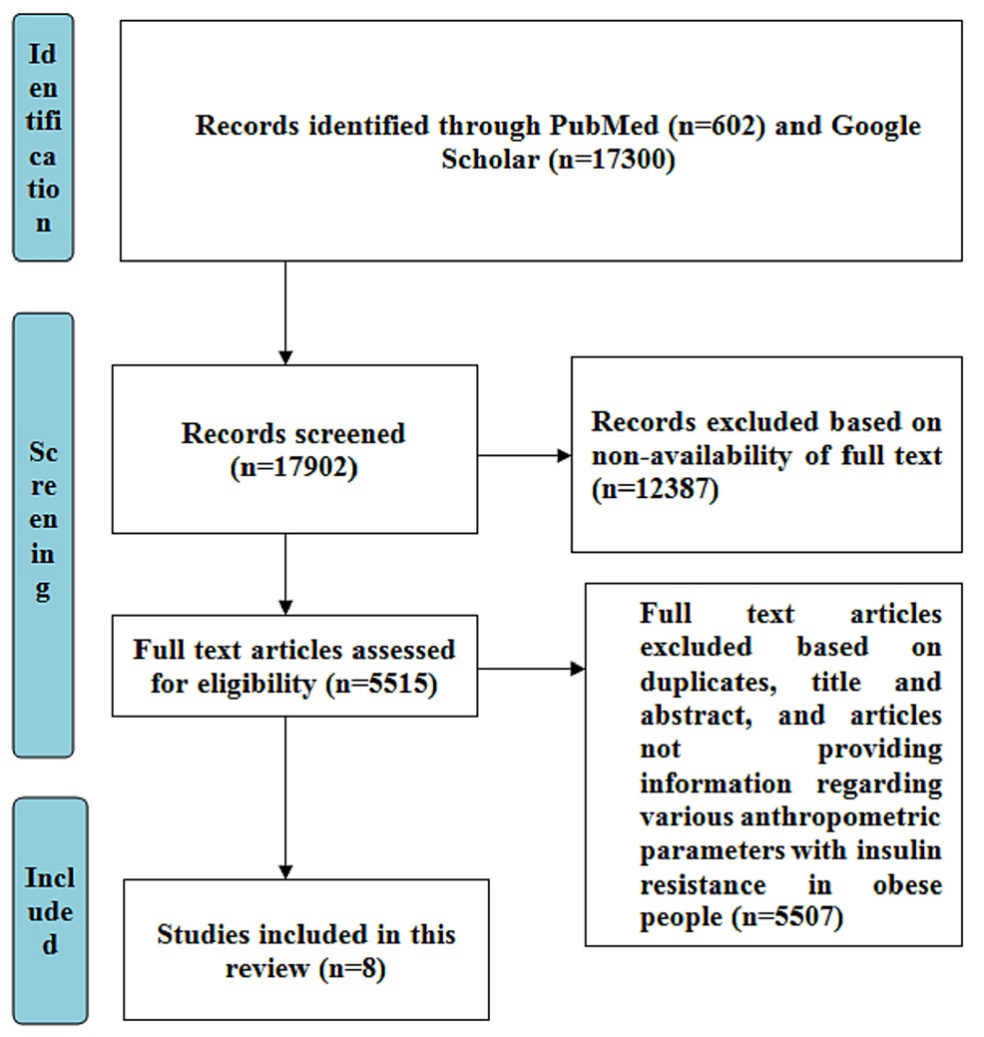
Search strategy

The search methodology involved searching PubMed and Google Scholar databases for articles that were published between 2018 and 2023. The keywords that were used to search the articles included “anthropometric parameters,” “insulin resistance,” and “obesity.” The inclusion criteria consisted of articles with full-text availability consisting of cross-sectional studies, articles comparing various anthropometric parameters with IR in obese people, studies done on humans, and articles in the English language. The articles not providing information regarding various anthropometric parameters with IR in obese people, having non-availability of full-text, and articles that were not published in the English language were excluded from the study. The articles were screened based on the inclusion and exclusion criteria and a total of 8 articles were included in this review. The search strategy was based on Preferred Reporting Items for Systematic Reviews and Meta-Analyses (PRISMA), as shown in Figure 1.

A review of all eight studies consisting of author and year, study design, methodology, and results is described in Table 1.

**Table 1:** Review of the studies BMI = Body mass index, TG = Triglyceride, TG/HDL = Triglyceride to high-density lipoprotein ratio, PCOS = Polycystic ovarian syndrome, WHR = Waist-to-hip ratio, FT3 = Serum-free triiodothyronine, FT4 = free thyroxine, TSH = Thyroid-stimulating hormone, FBS = Fasting blood sugar, HOMA-IR = Homeostatic model assessment of insulin resistance, DEXA = Dual-energy X-ray absorptiometry, MRI = Magnetic resonance imaging, ROC = Receiver operating characteristic (ROC) curves, CUN-BAE = Clinica Universidad de Navarra-body adiposity estimator, MetS = Metabolic syndrome, HDL = High-density lipoprotein, CES-D = Centre for Epidemiological Studies-Depression, WC = Waist circumference, NC = neck circumference, HC = Hip circumference, CVD = cerebrovascular disease, WHtR = waist-to-height ratio, TFM = Trunk fat mass, %TFM = Percent trunk fat mass, WBTFM = Whole-body total fat mass, %WBTFM = Percent whole-body total fat mass.

Sr. No.	Author and Year	Study Design	Methodology	Findings
1.	Elrayess et al., 2020 [18]	Cross-sectional study	On the campus of Qatar University, 150 sound young ladies with BMIs ranging from 24.3 to 5.3 kg/m^2^ were sought out. To examine the incidence and regulators of insulin resistance in age-matched groups, the research focused on college students.	In this research, we emphasized the elevated rate of insulin resistance in excessive-weight, young Qatari girls, and we found that TG and TG/HDL ratios were significant predictors of insulin resistance in this population.
2.	Venkatesh et al., 2022 [19]	A Mendelian randomization study	Data on obesity and reproductive disorders for a total of 257,193 women of European ancestry were subjected to Mendelian randomization, generalized additive models, and logistic regression analysis. Uterine fibroids, PCOS, heavy monthly bleeding, and pre-eclampsia were all seen to be genetically and statistically linked with BMI, WHR, and WHR corrected for the BMI.	Frequently used indicators of overall and central obesity were connected to greater chances of reproductive anomalies to varying degrees in a comprehensive, extensive, genetics-based analysis of the causative linkages among obesity and female reproductive issues.
3.	Du et al., 2019 [20]	Randomized clinical trial	500 non-obese individuals and 858 centrally obese patients were chosen at random for this study. Serum-free triiodothyronine (FT3), free thyroxine (FT4), thyroid-stimulating hormone (TSH), BMI, WHR, FBS and insulin HOMA-IR, lipid concentrations, and blood pressure were all determined for every single participant. The Centre for Epidemiological Studies-Depression (CES-D) scale was used to measure depression.	The study discovered significant rates of sadness and hypothyroidism among people who were centrally fat. To regulate weight, FT4 and TSH are crucial. Obesity and depression have an advantageous relationship.
4.	Bravo et al., 2018 [21]	Literature review	Obtaining significant published papers in the domains taken into consideration by this research required the utilization of two bibliographic databases, as well as the bibliographic references of all assigned articles.	The study draws the conclusion that the anthropometric studies under consideration had not given much thought to the investigation of the accuracy, reliability, and perfection of the manual measuring methods. Therefore, anthropometric research should focus greater attention and effort on assessing an inaccurate measurement and specifying the techniques used to gather anthropometric data in order to prevent assessment mistakes and inaccurate outcomes.
5.	Lee et al., 2013 [22]	A randomized clinical trial	Boys in their teen years who are overweight were sought. The participants had to be between the ages of 12 and 18, pubertal, nonsmokers, not diabetic, and inactive physically. The evaluation of pubertal growth, physical evaluation, and full medical history was done.	Dual-energy X-ray absorptiometry (DEXA) tends to overestimate adipose reductions and skeletal muscle growth in comparison to MRI due to systematic errors in the estimates between the modalities. So, the approach used has an impact on the changes that have been noticed in physical makeup.
6.	Sekgala et al., 2022 [23]	Cross-sectional study	Cross-sectional research, including 185 male cab drivers, was carried out. We assessed their height, weight, WC, and blood lipid profile. The effectiveness of anthropometric variables to predict metabolic syndrome detection was compared using receiver operating characteristic (ROC) curves.	While the percentage body fat, body roundness index, conicity index, body mass index, and Clnica Universidad de Navarra-body adiposity estimator (CUN-BAE) could predict MetS among South African male taxi drivers, when predicting the individual MetS risk signs, these variables performed less well.
7.	Wan et al., 2020 [24]	Cross-sectional study	In 2018, a total of 4,658 diabetic individuals from seven Chinese villages were registered. In addition to filling out questionnaires, individuals had their blood pressure, glucose, lipid profile, urine albumin/creatinine ratio, fundus photos, and anthropometric measures of their height, weight, WC, NC, and HC taken.	The research shows that, among the abdominal obesity indices, the Chinese visceral adiposity index showed the greatest correlation with the incidence of cerebrovascular disease (CVD) and diabetic kidney problems, and NC had a distinctive correlation with incidences of CCA plaque. NC could be a practical as well as useful anthropometric parameter for CVD early detection.
8.	Lee et al., 2021 [25]	Cross-sectional survey	In this cross-sectional study, complex-samples multiple logistic regression models using complex-sample survey data were used to compare the relationships between BMI, WHtR, WC and TFM, %TFM, WBTFM, and %WBTFM as body composition indices with metabolic risk factors.	Due to anthropometric parameters may have an equivalent or greater ability to detect metabolic risk markers than physical composition metrics, their usage is not appropriate for the massive operations testing of the adult Korean population for metabolic disorders. Further, as in comparison with other variables in the Korean population, WHtR was comparable to or more linked with diabetes, hypertriglyceridemia, hyperlipidemia, and in men and hypertension, diabetes, hypo-HDL cholesterolemia, and hypo-HDL cholesterolemia in women.

Discussion

By promoting molecular consumption, the hormone insulin regulates the metabolism of carbs, proteins, and lipids. There are various factors that alter the sensitivity to insulin and also contribute to IR. IR is regarded as the pathogenic operator for numerous nowadays illnesses, including atherosclerosis, non-alcoholic fatty liver disease (NAFLD), metabolic syndrome, and type 2 diabetes (type 2 DM). When insulin-targeting tissues are less reactive to elevated physiological insulin levels, this is referred to as IR [26, 27]. According to research, those with IR gained less weight than people with insulin sensitivity [2]. So, it is very essential to know about the role of insulin and the problems caused by IR.

Obesity is described as an excessive or unusual buildup of fat in adipose tissue, which compromises a person's well-being [3-5]. It is a frequently occurring curable illness of clinical and public health value. Due to a growing sedentary lifestyle of many occupations, high-calorie, high-fat diets, shifting methods of travel, and growing urbanization, obesity is brought on [5]. Hence, it is very important to know the cause of obesity and diagnose it as soon as possible by using appropriate anthropometric assessment tools, which among the measurements used in the investigation could most accurately foresee the dangers of obesity is hard to determine with compliance, but it can be determined that some of the recently validated anthropometric adiposity markers could be used to determine clinical circumstances after additional verification [28].

On the basis of various studies, we can conclude that there are a number of anthropometric parameters available with variability in specificity and reliability to evaluate the obesity and risk parameters linked with obesity. The parameters such as BMI, WC, WHR, WHtR, AVI, BAI, BRI, and ABSI can be used for evaluation purposes. BMI was not the best anthropometric characteristic for determining the possibility of chronic heart disease, according to research. Additionally, ABSI was the strongest indicator for men, whereas the top parameters for women were WHtR and BRI [14]. Goh et al. in their research found that WC, WHR, and WSR were better indices of central obesity and showed a stronger correlation with traditional CVD risk variables. Additionally, they discovered that compared to overall obesity, central obesity had a stronger association with CVD risk. Because it could not identify between fat and fat-free mass, BMI itself was insufficient for recognizing people with a greater likelihood of CVD. They also mentioned that anthropometric parameters of central obesity had great sensitivity and specificity [8]. Sánchez-Garcia et al. in their study mentioned that WC was more in elderly women than elderly men. They also mentioned that BMI estimation alone was associated with overestimation in the overweight elderly population [9].

For the classification of obesity, an estimation of body fat content is required. Underwater weighing, CT, MRI, and DEXA scanning are the methods that accurately assess body fat [29]. However, they are not appropriate for use in routine clinical practice. BMI and WC, which have restrictions relative to these imaging technologies but nonetheless give pertinent data and are simple to employ in a number of practice environments, are other ways of estimating body fat.

Kamadjeu et al. in their study found that WC was more predictive of visceral fat which had more deleterious effects on health [10]. They came to the conclusion that WC is a useful indicator of intra-abdominal fat accumulation and should be used to pinpoint individuals who require treatment to lower their likelihood of cardiovascular disease. They found that WC had a strong relationship with BMI and a moderate relationship with WHR. BMI measured total body fat, while WC and WHR measured central obesity. BMI, WC, and WHR all are lower in young males than females [10].

Ben-Noun et al. in their study found that NC exhibited a strong inverse relationship with BMI, WC, WHR, total cholesterol, low-density lipoprotein-cholesterol, TG, glucose level, uric acid, systolic blood pressure (SBP), and diastolic blood pressure (DBP) [30]. Li et al. in their study found that NC and visceral adipose tissue (VAT) are favorably associated in both sexes. BMI, WC, and WHtR are substantially linked with VAT in both men and women. In both sexes, BMI had a stronger correlation with VAT than NC. WHR showed the least strong association with VAT in males and no link in women [31].

Iwani et al. also observed that an increased TG/HDL ratio is linked with IR and compared it with HOMA-IR [32]. Apart from its previously known specific relationship with LDL, Kohli et al. in their investigation showed that the TG/HDL-C ratio was highly correlated with other lipid variables and markers of adiposity, such as BMI and body fat [33]. Cordero et al. also mentioned that TG/HDL ratio values >2.75 in men and >1.65 in women were found in the study of metabolic syndrome in active subjects (MESYAS) [34]. Wang et al. in their study used eight anthropometric parameters (BMI, AVI, BAI, WHR, WHtR, BRI, WC, and ABSI) for assessing obesity and risk of CVD. They claimed that BMI was not the best anthropometric variable to gauge the likelihood of CHD. Whereas WHtR and BRI were the top indicators for ladies, ABSI was the greatest predictor for men [14].

According to Chen et al., QUICKI and log (HOMA) are two of the most reliable and practical surrogate measures for assessing insulin sensitivity in people [35]. Singh et al. mentioned various indices of IR, their advantages and disadvantages, and comparison with each other concluding that the most effective and most widely verified surrogates that can provide a better physiological measure of glucose homeostasis are HOMA and QUICKI [36]. As it is a manual assessment tool, it may show variations in the outcome even by using the same methodology, which defines less specificity towards evaluation of the obesity by using anthropometric parameters.

The primary goal of the research is to know about anthropometric parameter changes in obese and non-obese individuals suffering from IR. The target of this research was to take a step toward analyzing obesity and its detection and management. Although the statistics seem promising, further research is necessary to fully comprehend this disorder. The present study supports future research to conduct randomized control trials to evaluate the individual anthropometric parameter’s accuracy and specificity, which will further help manage this condition more effectively and accurately.

## Conclusions

The review demonstrated that BMI and WC were the most commonly used anthropometric parameters in routine practices due to their easy accessibility. Additionally, variations in the value of the parameters were also observed due to gender differences. Hence, in conclusion, as the prevalence of obesity cases is rising due to sedentary lifestyles, unhealthy food habits, lack of physical activity, and lack of awareness among young adults, early detection through various anthropometric measurement parameters and adequate and appropriate management is the need of the hour.
